# Vertical Gate-All-Around Device Architecture to Improve the Device Performance for Sub-5-nm Technology

**DOI:** 10.3390/mi13091551

**Published:** 2022-09-19

**Authors:** Changwoo Noh, Changwoo Han, Sang Min Won, Changhwan Shin

**Affiliations:** 1 Department of Semiconductor and Display Engineering, Sungkyunkwan University, Suwon 16419, Korea; 2 Semiconductor R&D Center, Samsung Electronics, Hwasung 18448, Korea; 3 Department of Electrical and Computer Engineering, Sungkyunkwan University, Suwon 16419, Korea; 4 School of Electrical Engineering, Korea University, Seoul 02841, Korea

**Keywords:** MOSFET, vertical gate-all-around, FinFET, nano-sheet FET, short channel effect

## Abstract

In this work, we propose a vertical gate-all-around device architecture (GAA-FinFET) with the aim of simultaneously improving device performance as well as addressing the short channel effect (SCE). The GAA-FinFET was built using the technology computer-aided design (TCAD) simulation tool, and then, its electrical characteristics were quantitatively evaluated. The electrical characteristics of the GAA-FinFET were compared to those of conventional FinFET and nano-sheet FET (NSFET) at 7 nm or 5 nm nodes. When comparing the GAA-FinFET against the FinFET, it achieved not only better SCE characteristics, but also higher on-state drive current due to its gate-all-around device structure. This helps to improve the ratio of effective drive current to off-state leakage current (i.e., I_eff_/I_off_) by ~30%, resulting in an improvement in DC device performance by ~10%. When comparing the GAA-FinFET against the NSFET, it exhibited SCE characteristics that were comparable or superior thanks to its improved sub-channel leakage suppression. It turned out that the proposed GAA-FinFET (compared to conventional FinFET at the 7 nm or 5 nm nodes, or even beyond) is an attractive option for improving device performance in terms of SCE and series resistance. Furthermore, it is expected that the device structure of GAA-FinFET is very similar to that of conventional FinFET, resulting in further improvement to its electrical characteristics as a result of its gate-all-around device structure without significant modification with respect to the processing steps for conventional FinFET.

## 1. Introduction

With the decline of planar bulk MOSFETs (metal–oxide–semiconductor field-effect transistors), fin-shaped field-effect transistors (FinFET) have been developed, making it possible to overcome physical limitations as well as achieving good electrostatic integrity in devices. This new 3D device structure has become the mainstream in the System LSI (large-scale integration) industry. However, as the physical dimensions of FinFET have been aggressively scaled down (e.g., shorter gate length, tighter fin-to-fin pitch, etc.), FinFET is faced with critical issues in terms of short channel control, device performance, and power consumption. To suppress short channel effects (SCEs), the fin width has become significantly more narrow. Considering the physical limitations presented by quantum mechanical issues in silicon channels, the narrowest possible fin width is about 5 nm. Moreover, it is necessary to increase the fin height in order to alleviate the leakage current around the bottom part of the fin. To improve device performance, the effective channel width of FinFET needs to be increased for a given layout area, by increasing fin height [1,2,3,4,5,6]. With increasing fin height, however, there is a corresponding increase in parasitic capacitance in the FinFET device structure. Therefore, although there is an improvement in the DC device performance of FinFET, the AC device performance does not improve, and can even deteriorate, due to the ever-increasing parasitic capacitance of FinFET with taller fin heights. For low-power applications, low power supply voltage (V_DD_) and low standby leakage current are necessary. In order to meet the required V_DD_ and leakage current specifications for low-power applications, superior gate-to-channel capacitive coupling in the subthreshold region as well as superior on-state drive current are required. Unfortunately, FinFET technology run out of steam [7,8,9,10], and novel device structures should be developed.

Gate-all-around (GAA) device structures can overcome the physical limitations faced by FinFET, and and demonstrate SCE characteristics and device performance. As one type of GAA device structure, nano-sheet FET (NSFET) has received a lot of attention because it can not only be sustainably scaled down, but also consumes less power and exhibits better electrical characteristics [8,9,10,11]. However, due to the different channel direction in NSFET (compared to FinFET), a modification in fabrication steps is necessary—the channel direction in FinFET is vertical, but the NSFET channel direction is horizontal [12,13] (e.g., replacement metal gate, channel formation and release, source/drain process, and so on).

Previous studies have compared FinFET against gate-all-around FET (GAA FET) in terms of many aspects, including device performance, power consumption, layout optimization, and so on. Many works have claimed that the GAA FET has superior device performance, e.g., better SCE, lower power consumption due to high gate controllability, and good design flexibility due to the wide possible range of variable nano-sheet widths [2,4,7,14]. However, there are technical challenges with respect to GAA FET that remain to be addressed, for example, (i) the gate-to-channel controllability of a bottom-side transistor for suppressing sub-threshold leakage as well as reducing parasitic capacitance, (ii) a strategy for providing multi-Vt with low Vt variation, (iii) design technology co-optimization (DTCO) for GAA FET, and (iv) production in high-volume manufacturing [15,16,17,18,19,20,21]. The GAA FET has the potential to continue being scaled down in order to meet the Moore’s Law, but there are technical problems to be addressed before it can be regarded as a next-generation device architecture following FinFET in the System LSI industry. Of course, there have been many works focusing on extending the use of FinFET technology for as long as possible in the field of sub-5 nm technology [22,23,24,25].

In this work, we propose a FinFET-based vertical gate-all-around device architecture (GAA-FinFET) with the aim of improving the device performance at sub-5 nm technology nodes. The channel of GAA-FinFET is surrounded by its gate, but the device structure looks similar to the device structure of conventional FinFET. It is expected that the electrical characteristics of GAA-FinFET can be improved due to its gate-all-around device structure without significantly modifying the process integration steps used for conventional FinFET. The GAA-FinFET was built using the technology computer-aided design (TCAD) simulation tool, and then its electrical characteristics were extracted. It turned out that the GAA-FinFET (compared to FinFET) is able to achieve not only better SCE characteristics, but also higher on-state drive current. In addition, the GAA-FinFET (compared to NSFET) demonstrates comparable or superior SCE characteristics thanks to its better sub-channel leakage suppression.

## 2. Device Structures and Calibration for Simulation

**Figure 1:**
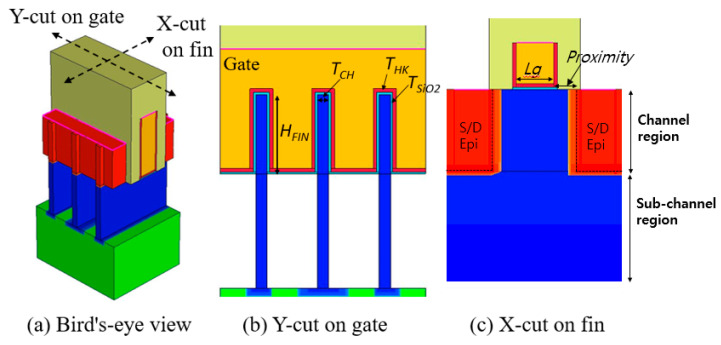
(**a**) Bird’s-eye and (**b**,**c**) cross-sectional views of FinFET. The major geometric device parameters are noted in the figures. Note that the fin shape is rectangular (not tapered). In addition, three fins are used in the channel region, and merged in epitaxially grown source/drain regions.

**Figure 2:**
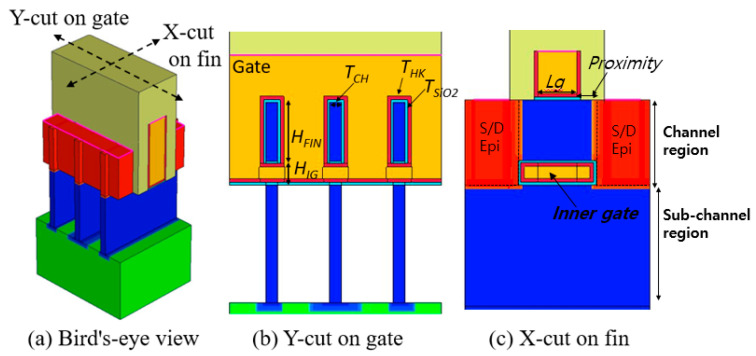
(**a**) Bird’s-eye and (**b**,**c**) cross-sectional views of FinFET-based vertical GAA-FinFET. The major geometric device parameters are noted in the figures. The device structure of the GAA-FinFET is similar to that of a FinFET device without an inner gate. The inner gate is placed on the bottom side of channel region. The length of the inner gate is defined as the gate length (L_g_) plus the proximity. The height of the inner gate (H_IG_) is 10 nm.

**Figure 3:**
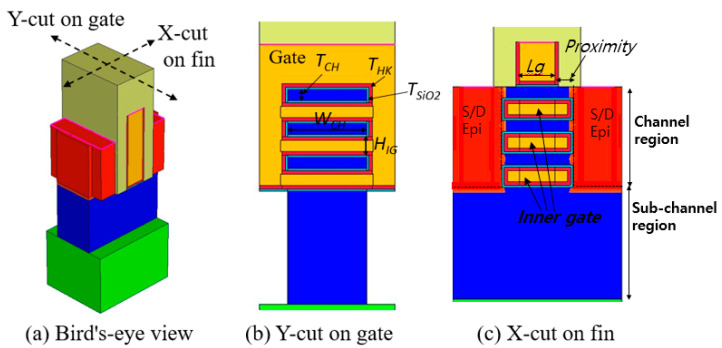
(**a**) Bird’s-eye and (**b**,**c**) cross-sectional views of nano-sheet FET (NSFET). The geometric device parameters are noted in the figures. The height of each inner gate (H_IG_) is 10 nm. The length of the inner gate is defined as the gate length (L_g_) plus the proximity. The channel width (W_CH_) of each nano-sheet is 35 nm.

Figure 1, Figure 2 and Figure 3 present a bird’s-eye view and cross-sectional views of FinFET, GAA-FinFET, and NSFET, respectively. The proximity is defined as the distance between the edge of the gate and the physical source/drain (S/D) epi layer, as shown in the X-cut on the fin (see Figure 1c). The HfO_2_ was used as the high-k material in the gate stack. For more details, the simulation parameters of each device are listed in Table 1.

As shown in Figure 1, the FinFET has three fins (the width and height of which are 5 nm and 35 nm, respectively) in its channel region. The depth of the S/D epi layer is designed to match the fin height. The fin shape is rectangular (i.e., not tapered) in order to achieve better suppression of sub-channel leakage current.

As shown in Figure 2, the device structure of GAA-FinFET is quite similar to that of the FinFET. The difference, however, lies in the placement of an inner gate on the bottom side of channel region. The inner gate is filled with a high-k material, an interface oxide layer, and a gate metal. The length of the inner gate is defined by “gate length + proximity × 2”. The height of the inner gate is 10 nm, and the fin height is 25 nm. The sum of the height of the inner gate (H_IG_) and the fin height of GAA-FinFET (H_FIN|GAA-FinFET_) is equal to the fin height of FinFET (H_FIN|FinFET_).

As shown in Figure 3, the NSFET has three nano-sheets and three inner gates in its channel region. The channel width of each nano-sheet is 35 nm. The length of the inner gate is defined by “gate length + proximity × 2”. The height of each inner gate is 10 nm.

**Figure 4:**
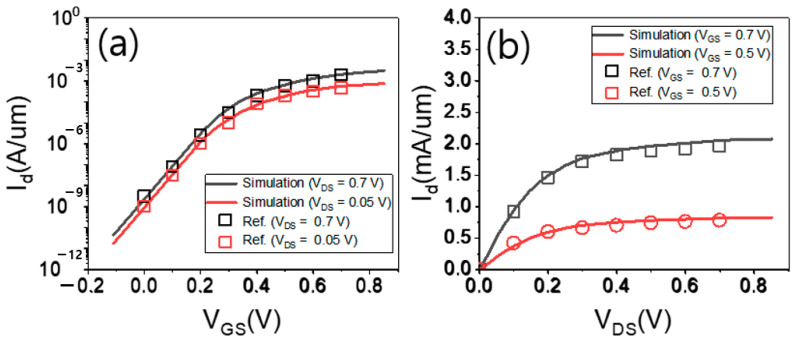
(**a**) Input and (**b**) output transfer characteristics of FinFET (which were calibrated to the measured data reported in [1]).

With calibration work, the simulated input/output transfer characteristics of FinFET were well matched to the measured data reported in [1] (see Figure 4). All devices were simulated using the Sentaurus TCAD tool. The bandgap narrowing model was used to consider the doping-dependent bandgap changes for all Si regions. The mobility models used in this work include (1) the inversion and accumulation layer model, (2) the thin-layer model, (3) the low-field ballistic mobility model, and (4) the Lombardi mobility model. The inversion and accumulation layer model and the thin-layer model were used for impurity, phonon, and surface roughness scatterings within a structural confinement of charge carriers in the thin channel. The low-field ballistic mobility model was used to capture the quasi-ballistic effects on the charge carrier mobility. The Lombardi mobility model was used for remote phonon and Coulomb scatterings. Recombination models included Shockley–Read–Hall, Auger, and band-to-band recombination models.

## 3. Results and Discussion

### 3.1. Comparison of Electrical Characteristics: FinFET vs. GAA-FinFET

**Figure 5:**
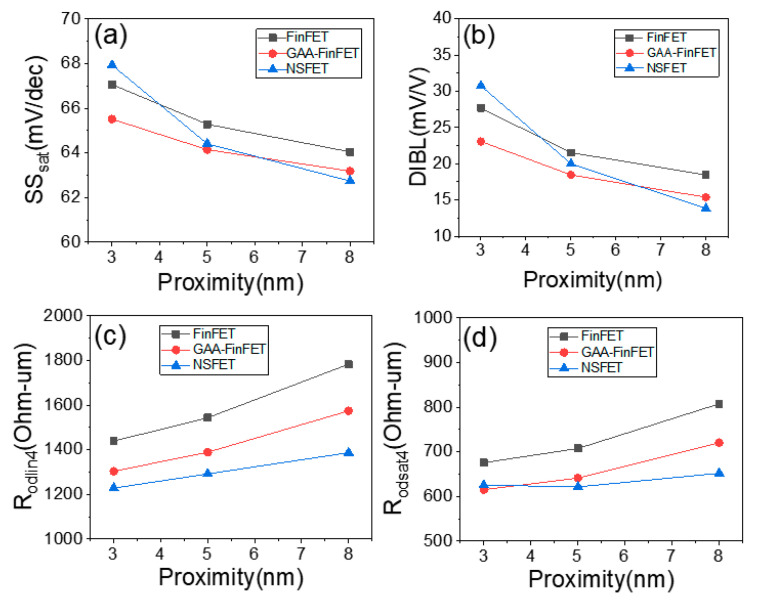
(**a**) SS_sat_, (**b**) DIBL, (**c**) R_odlin4_, and (**d**) R_odsat4_ for three values of proximity (i.e., 8 nm, 5 nm, and 3 nm). Please note that the doping concentration in the sub-channel region is set to 1 × 10^18^ cm^−3^.

As shown in Figure 5a,b, the GAA-FinFET is superior to the FinFET in terms of short-channel-effect (SCE) immunity for the tested proximity values (from 3 nm to 8 nm). At a proximity of 8 nm, the subthreshold swing (SS_sat_) and drain-induced barrier lowering (DIBL) of GAA-FinFET (compared to FinFET) were improved from 64.0 mV/decade to 63.2 mV/decade and from 18.5 mV/V to 15.4 mV/V, respectively. With increasing proximity (i.e., with decreasing distance), SS_sat_ and DIBL were further improved. At a proximity of 3 nm, SS_sat_ and DIBL improved from 67.1 mV/decade to 65.5 mV/decade and from 27.7 mV/V to 23.1 mV/V, respectively. This indicates that the GAA-FinFET (compared to conventional FinFET) is better able to suppress SCE, and the SCE of GAA-FinFET (compared to FinFET) is less degraded for the aggressively scaled-down distance between the edges of the source and the drain epi layer, even though the SCE of those two devices should be worse at shorter distances. The gate-to-channel capacitive coupling was enhanced due to the GAA device structure (i.e., the total area controlled by the gate electrode is wider in GAA-FinFET than in FinFET). This effectively increases the “Cox”, resulting in steeper SS_sat_, as shown in Equation (1) [26]. Moreover, sub-channel leakage current can be further suppressed by its inner gate (note that the FinFET does not have its own inner gate).

(1) $$\;\mathrm{SS}={\left[\frac{d\left(log_{10}\;I_{D}\right)}{dV_{G}}\right]}^{-1}=2.3\;\frac{K_{B}T}{e}\left(\frac{C_{ox}+C_{dep}+C_{it}}{C_{ox}}\right)$$

**Figure 6:**
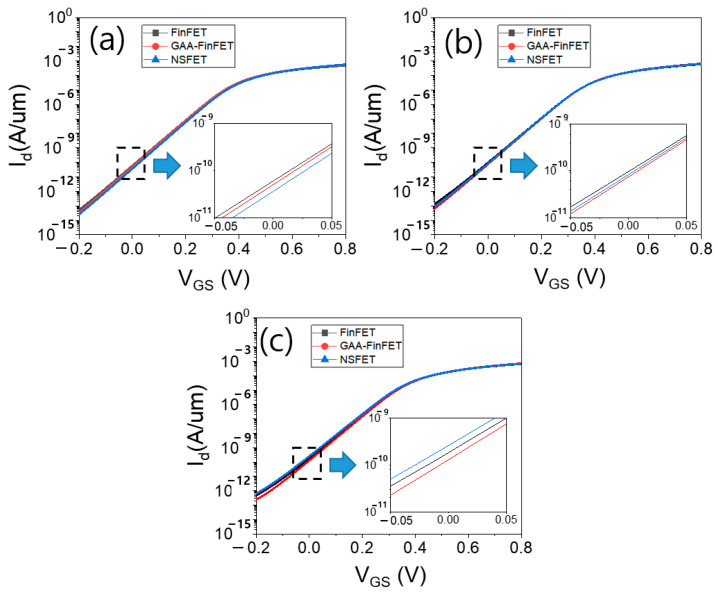
The input transfer characteristics of three devices (V_DS_ = 0.7 V) for proximities of (**a**) 8 nm, (**b**) 5 nm, and (**c**) 3 nm. Please note that the doping concentration in the sub-channel region is set to 1 × 10^18^ cm^−3^.

Figure 6a–c show the input transfer characteristics of the three devices with a power supply voltage of 0.7 V for given proximities of 8 nm, 5 nm, and 3 nm, respectively. With increasing proximity (i.e., with decreasing distance), the off-state leakage current (i.e., Id at V_GS_ of 0 V) of all devices increased. However, the off-state leakage current of GAA-FinFET showed a smaller increase than that of FinFET and NSFET.

Investigating the R_odlin4_ and R_odsat4_ parameters, the resistance characteristics of GAA-FinFET vs. FinFET are compared to each other. Please note that R_odlin4_ and R_odsat4_ indicate the total resistance between the source and the drain (calculated on the basis of the value of V_DS_ / I_DS_ for a given gate voltage of V_TH_ + 0.4 V) when the transistor is operating in linear mode (V_DS_ = 0.05 V) and saturation mode (V_DS_ = 0.7 V), respectively. The bias condition is as follows: (V_GS_, V_DS_) = (V_TH_ + 0.4 V, 0.05 V) for R_odlin4_, and (V_GS_, V_DS_) = (V_TH_ + 0.4 V, 0.7 V) for R_odsat4_. The reason for plugging the value “V_TH_ + 0.4 V” into the term for V_GS_ is to be able to measure I_DS_ without it being impacted by the V_TH_ of the devices. In other words, by using R_odlin4_ and R_odsat4_, the resistance characteristics can be compared with each other without being impacted by differences in V_TH_. These values are normalized to the effective channel width of each device. As shown in Figure 5c,d, the GAA-FinFET is superior to the FinFET in terms of resistance for the tested proximity values (i.e., from 3 nm to 8 nm). At a proximity of 8 nm, the R_odlin4_ and R_odsat4_ of GAA-FinFET (compared to FinFET) improve from 1783 ohm-um to 1574 ohm-um, and from 808 ohm-um to 721 ohm-um, respectively. At a proximity of 3 nm, R_odlin4_ and R_odsat4_ improve from 1439 ohm-um to 1304 ohm-um, and from 676 ohm-um to 615 ohm-um, respectively. This indicates that the GAA-FinFET (compared to FinFET) has better resistance characteristics when operating in both linear and saturation mode.

**Figure 7:**
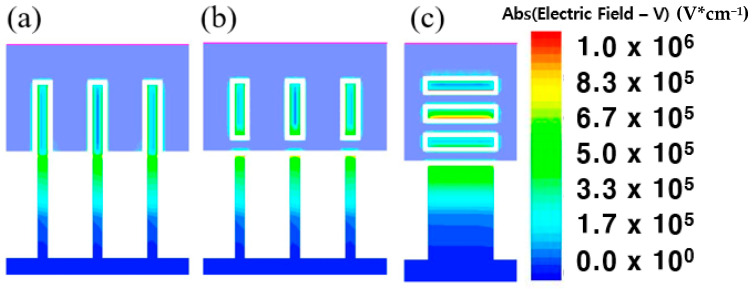
Cross-sectional views of electrical field density in (**a**) FinFET, (**b**) GAA-FinFET, and (**c**) NSFET.

Figure 7a,b show a cross sectional view of an electrical field density of FinFET and GAA-FinFET, respectively. It can be seen that a channel near the inner gate (tri-gate region) of GAA-FinFET has a higher electrical field density than the middle part of the channel (double-gate region). More specifically, the GAA FinFET has its own “inner gate”, increasing the total gate area wrapping around the channel region. (1) This should induce greater charge in the channel for a given voltage (note that Q = CV). (2) This should help to enhance the electric field density, because the corner regions on the top and bottom sides of the channel region are more exposed to the gate electrodes, intensifying the electric field density. These two phenomena result in a decrease in total resistance.

**Figure 8:**
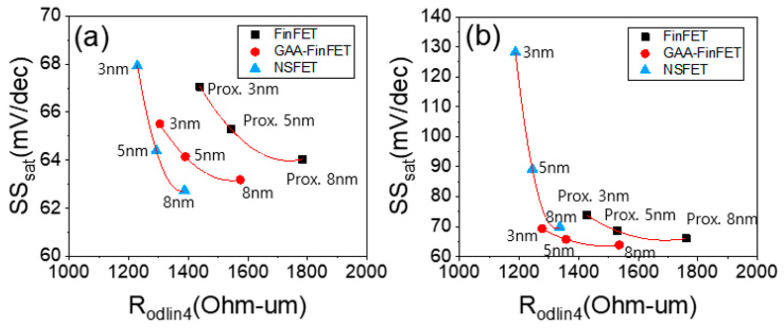
Trade-off between SS_sat_ and R_odlin4_ at three values of proximity (i.e., 8 nm, 5 nm, and 3 nm) in each device. The doping concentration in the sub-channel region was set to (**a**) 1 × 10^18^ cm^−3^ and (**b**) 5 × 10^17^ cm^−3^.

Figure 8a shows SS_sat_ vs. R_odlin4_ at three values of proximity. This shows the trade-off between SCE and resistance characteristics with variations in proximity. In general, with increasing proximity, the resistance characteristics improve, but the SCE characteristics become worse due to the shorter effective length. The GAA-FinFET (compared to FinFET) is able to achieve superior SS_sat_ with lower resistance (R_odlin4_), especially when a lower doping concentration in the sub-channel region is used (see Figure 8a vs. Figure 8b; further details will be discussed in Section 3.3).

**Figure 9:**
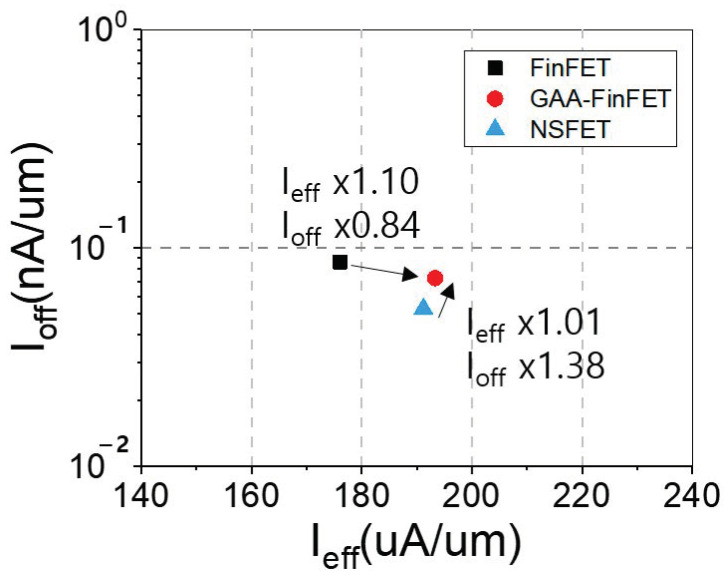
Off-state leakage current (I_off_) versus effective drive current (I_eff_) for FinFET and GAA-FinFET. Please note that the currents are normalized to the effective channel width.

**Figure 10:**
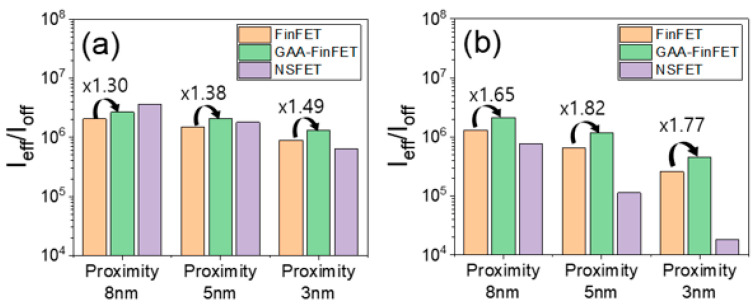
I_eff_/I_off_ for FinFET, GAA-FinFET, and NSFET. Three proximity values (i.e., 8 nm, 5 nm, and 3 nm) were used at two different doping concentrations in the sub-channel region: (**a**) 1 × 10^18^ cm^−3^ and (**b**) 5 × 10^17^ cm^−3^.

Figure 9 and Figure 10a show off-state leakage current (I_off_) vs. effective drive current (I_eff_) and the ratio of effective drive current to off-state leakage current (i.e., I_eff_/I_off_), respectively. The I_eff_ of GAA-FinFET (compared to FinFET) demonstrates an improvement of ~10%, and the I_eff_/I_off_ is improved by ~30%. Furthermore, with increasing proximity (i.e., from 8 nm to 3 nm), the degree of improvement of I_eff_/I_off_ of GAA-FinFET (compared to FinFET) increases to its better immunity to SCE and lower resistance.

### 3.2. Comparison of Electrical Characteristics: NSFET vs. GAA-FinFET

At a proximity of 8 nm, NSFET is superior to GAA-FinFET in terms of SCE immunity (see Figure 5a,b). However, at proximities of 5 nm and 3 nm, GAA-FinFET is better than NSFET. This is based on the observation that, with increasing proximity, the degradation of the SCE of NSFET is more intense as a result of the bottom-side transistor of NSFET in the sub-channel region. The device structure of the bottom-side transistor of NSFET looks similar to a planar MOSFET structure, so that the channel width of the bottom-side transistor is not narrow enough to suppress sub-channel leakage current. In other words, the bottom-side transistor of NSFET is highly vulnerable to SCE. On the other hand, with increasing proximity, the degradation of the SCE of GAA-FinFET (compared to NSFET) takes place to a lesser extent, because the GAA-FinFET has its own narrow fin for suppressing sub-channel leakage current.

As shown in Figure 5c,d, the NSFET is superior to GAA-FinFET in terms of resistance except for one case—saturation mode at a proximity of 3 nm. It can be seen that the channel of NSFET, especially the middle one of the three nano-sheets, has a higher electrical field density than the other channel of GAA-FinFET (see Figure 7b,c). The physical reason for the improved resistance characteristics of NSFET is the increased electrical field density in the middle channel of NSFET.

As shown in Figure 10a, the I_eff_/I_off_ of NSFET (compared to GAA-FinFET) is higher only for the proximity of 8 nm. This is because, with increasing proximity, the I_off_ of NSFET increases significantly due to the degradation of SCE. However, the I_eff_/I_off_ of NSFET (compared to GAA-FinFET) is lower at all the proximities with lower doping concentration in the sub-channel region (see Figure 10a vs. Figure 10b, and more details will be discussed in Section 3.3).

### 3.3. Impact of Doping Concentration in the Sub-Channel Region on Electrical Characteristics

In the previous simulations shown/discussed in Section 3.1 and Section 3.2, sub-channel leakage current was well suppressed with using heavy doping profile in the sub-channel region. Herein, to investigate the impact of doping concentration in the sub-channel region on the electrical characteristics, a lower doping concentration in the sub-channel region (i.e., from 1 × 10^18^ cm^−3^ to 5 × 10^17^ cm^−3^) is, on purpose, used to have the sub-channel leakage current to be incompletely suppressed. When the doping concentration in the sub-channel region becomes lighter, the depletion width near source/drain epi junction region becomes wider. This causes the distance between the depletion region of source and that of drain to be narrower, so that the leakage current through the sub-channel region increases by punch-through event.

**Figure 11:**
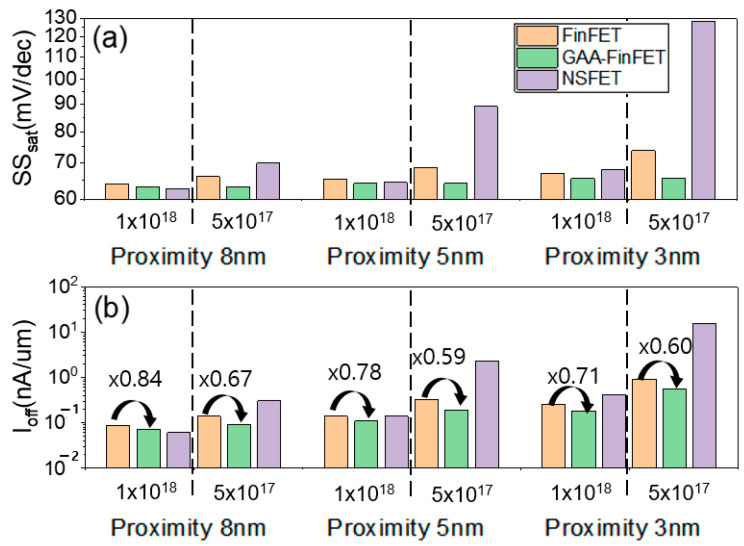
(**a**) SS_sat_ and (**b**) I_off_ for FinFET, GAA-FinFET, and NSFET. Three values of proximity (i.e., 8 nm, 5 nm, and 3 nm) were used for two different doping concentrations in the sub-channel region: 1 × 10^18^ cm^−3^ and 5 × 10^17^ cm^−3^.

For both the FinFET and NSFET, the SCE characteristics for the given proximity values (i.e., from 3 nm to 8 nm) (see Figure 11a) were degraded because the lower doping concentration is not enough to suppress sub-channel leakage current in the device structure. For the GAA-FinFET, however, the SCE characteristics were not degraded even with a proximity of 3 nm. In other words, it turned out that the device structure of GAA-FinFET (compared to that of FinFET and NSFET) was more robust to sub-channel leakage current. The GAA-FinFET in reality has a fin in the sub-channel region, which is sufficiently narrow to suppress sub-channel leakage current. In addition, it has superior gate controllability in the sub-channel region because the inner gate of GAA-FinFET covers one side of the sub-channel (note that there is no gate covering the sub-channel in the FinFET device structure). Compared to NSFET, the GAA-FinFET has a narrow fin width in order to suppress sub-channel leakage current, rather than the wide channel width of NSFET, thus giving it the lowest off-state leakage current (I_off_) when lower doping concentrations in the sub-channel region are used (even at lower doping concentrations in the sub-channel region, i.e., 5 × 10^17^ cm^−3^) (see Figure 11b). The degree of improvement of I_off_ for the GAA-FinFET (compared to FinFET) was greater (i.e., from 16~29% to 33~41%).

**Figure 12:**
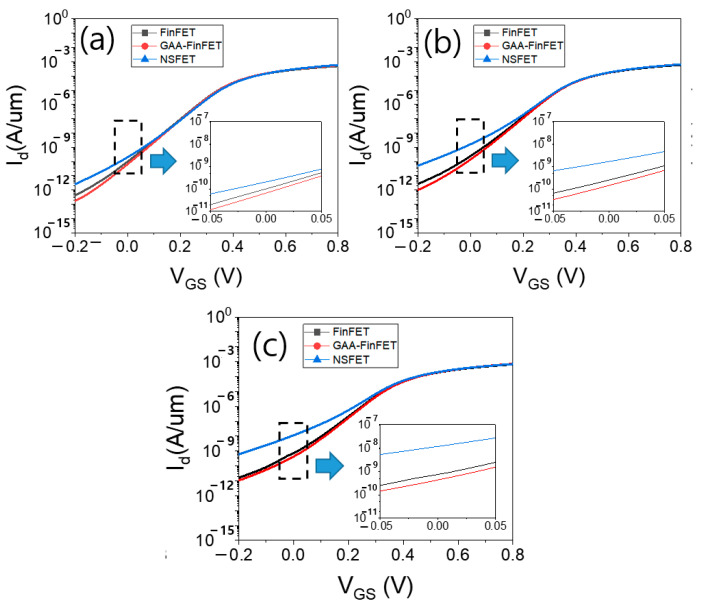
The input transfer characteristics of the three devices (V_DS_ = 0.7 V) for proximities of (**a**) 8 nm, (**b**) 5 nm, and (**c**) 3 nm. Please note that the doping concentration in the sub-channel region is set to 5 × 10^17^ cm^−3^.

Figure 12a–c show the input transfer characteristics of three devices with power supply voltage of 0.7 V for the given proximities of 8 nm, 5 nm, and 3 nm, respectively. As in the results obtained with a doping concentration of 1 × 10^18^ cm^−3^, the off-state leakage current (I_d_ at V_GS_ of 0 V) of GAA-FinFET exhibited a smaller decrease than that obtained for FinFET and NSFET with increasing proximity.

With a lower doping concentration in the sub-channel region (i.e., from 1 × 10^18^ cm^−3^ to 5 × 10^17^ cm^−3^) (see Figure 10b), the highest I_eff_/I_off_ was achieved by the GAA-FinFET (in comparison to FinFET, and NSFET). This is because the GAA-FinFET has low off-state leakage current due to its narrow fin in the sub-channel region and its gate-all-around device structure.

## 4. Conclusions

In this work, we propose a vertical gate-all-around FinFET device (GAA-FinFET) with the aim of improving device performance. The GAA-FinFET was built using the TCAD simulation tool, and then, its electrical characteristics were extracted. In comparison to FinFET, the GAA-FinFET exhibited not only better SCE characteristics, but also higher on-state drive current as a result of its gate-all-around device structure. This helps to improve I_eff_/I_off_ by ~30%, leading to an improvement in DC device performance by ~10%. In comparison to NSFET, the GAA-FinFET exhibited superior SCE characteristics thanks to the improved sub-channel leakage suppression by the narrow fin. It turned out that the proposed GAA-FinFET (compared to conventional FinFET) can be employed as a knob for suppressing SCE as well as for improving series resistance. Furthermore, while the device structure of GAA-FinFET is very similar to that of conventional FinFET, further gains/improvements in electrical characteristics were able to be obtained due to its gate-all-around device structure without significant modification to the FinFET fabrication process.

## Figures and Tables

**Table 1:** Simulation parameters of the device structures in this work.

Symbol	Parameters	FinFET	GAA-FinFET	NSFET
L_g_	Gate length	16 nm	16 nm	16 nm
H_FIN_ (W_CH_)	Fin height (Channel width)	35 nm	25 nm	35 nm
FP	Fin pitch	27 nm	27 nm	N/A
T_CH_	Channel thickness	5 nm	5 nm	5 nm
H_IG_	Inner gate height	N/A	10 nm	10 nm
-	S/D epi doping concentration	10^20^ cm^−3^ (Arsenic)	10^20^ cm^−3^ (Arsenic)	10^20^ cm^−3^ (Arsenic)
-	Channel region doping concentration	5 × 10^17^ cm^−3^ (Boron)	5 × 10^17^ cm^−3^ (Boron)	5 × 10^17^ cm^−3^ (Boron)
T_HK_	Gate high-k thickness	1.5 nm	1.5 nm	1.5 nm
T_SiO2_	Gate oxide layer thickness	1 nm	1 nm	1 nm

## Data Availability

The data presented in this study are available on request from the corresponding author.
